# Modulation of soluble guanylate cyclase ameliorates pulmonary hypertension in a rat model of chronic thromboembolic pulmonary hypertension by stimulating angiogenesis

**DOI:** 10.14814/phy2.15156

**Published:** 2022-01-10

**Authors:** John Zagorski, Evandro Neto‐Neves, Nathan J. Alves, Amanda J. Fisher, Jeffrey A. Kline

**Affiliations:** ^1^ Department of Emergency Medicine Indiana University School of Medicine Indianapolis Indiana USA; ^2^ Department of Anesthesia Indiana University School of Medicine Indianapolis Indiana USA; ^3^ Present address: Department of Medicine Indiana University School of Medicine Riley R2 435, 950 W. Walnut St. Indianapolis Indiana 46202 USA; ^4^ Present address: Department of Pharmacology Riberiao Proto Medical School University of San Paulo Sau Paulo Brazil

**Keywords:** angiogenesis, cinaciguat, CTEPH, guanylate cyclase

## Abstract

Acute pulmonary embolism (PE) does not always resolve after treatment and can progress to chronic thromboembolic disease (CTED) or the more severe chronic thromboembolic pulmonary hypertension (CTEPH). The mechanisms surrounding the likelihood of PE resolution or progress to CTED/CTEPH remain largely unknown. We have developed a rat model of CTEPH that closely resembles the human disease in terms of hemodynamics and cardiac manifestations. Embolization of rats with polystyrene microspheres followed by suppression of angiogenesis with the inhibitor of vascular endothelial growth factor receptor 2 (VEGF‐R2) SU5416 results in transient, acute pulmonary hypertension that progresses into chronic PE with PH with sustained right ventricular systolic pressures exceeding 70 mmHg (chronic pulmonary embolism [CPE] model). This model is similar to the widely utilized hypoxia/SU5416 model with the exception that the “first hit” is PE. Rats with CPE have impaired right heart function characterized by reduced VO_2_Max, reduced cardiac output, and increased Fulton index. None of these metrics are adversely affected by PE alone. Contrast‐mediated CT imaging of lungs from rats with PE minus SU5416 show large increases in pulmonary vascular volume, presumably due to an angiogenic response to acute PE/PH. Co‐treatment with SU5416 suppresses angiogenesis and produces the CTEPH‐like phenotype. We report here that treatment of CPE rats with agonists for soluble guanylate cyclase, a source of cGMP which is in turn a signal for angiogenesis, markedly increases angiogenesis in lungs, and ameliorates the cardiac deficiencies in the CPE model. These results have implications for future development of therapies for human CTEPH.

## Graphical abstract

Pulmonary embolism (PE) results increased pulmonary vascular angiogenesis. Blocking endogenous angiogenesis with the vascular endothelial growth factor receptor inhibitor SU5416 causes chronic progressive pulmonary hypertension. Stimulating angiogenesis with the soluble guanylate cyclase agonist cinaciguat or riociguat ameliorates chronic PE/hypertension.

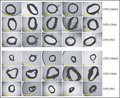

## 1 INTRODUCTION

Each year, approximately 600,000 persons in the US have PE diagnosed, and 30,000 die, making PE the third most common cause of cardiovascular death in the US (AHRQ Quality, 2018; Barco et al., 2020; Mozaffarian et al., 2015). The 30‐day case fatality rate for PE remains approximately 6%–8% overall and for severe PE (any arterial hypotension), the case fatality rate increases to 35% (Alotaibi et al., 2016; Spencer et al., 2010; Stein et al., 2012). Patients with PE that causes acute PH are at higher risk of RV failure and death within a few days (Jaff et al., 2011; Konstantinides et al., 2014). Treatment options for patients with more severe PE who are more likely to progress to PH remain limited to the acute use of systemic or catheter‐delivered fibrinolytic agents, which increase bleeding risk without a clear mortality improvement or benefit to RV function (Chatterjee et al., 2014; Nakamura et al., 2014; Riera‐Mestre et al., 2014; Tafur et al., 2017).

About 30% of patients with PE go on to have chronic thromboembolic disease (CTED), which in turn manifests a clinical spectrum, ranging from mild exercise‐induced fatigue and dyspnea (post‐PE syndrome) (Klok, van der Hulle, et al., 2016) to devastating chronic thromboembolic pulmonary hypertension (CTEPH) (Fernandes et al., 2016; Held et al., 2016; Kline et al., 2017; Sista et al., 2017). CTED appears to result from a combination of factors, including hypofibrinolysis and recurrent PE (Lang et al., 2008; Fernandes et al., 2016). Although understanding of the progression of CTED is poor, current data suggest about 10% of patients have progressive PH and develop the full blown CTEPH syndrome, defined by persistent perfusion defects and a resting mPAP>35 mmHg with LVEDP <15 mmHg (Nijkeuter et al., 2007; Pepke‐Zaba et al., 2011). At present, the only FDA‐approved drug to treat CTEPH is the soluble guanylate cyclase (sGC) stimulator, riociguat (RIO) (Dasgupta et al., 2015). Currently, RIO is administered after CTEPH diagnosis and is thought to work by causing pulmonary arterial smooth muscle relaxation.

It is unclear why only a fraction of patients with equal degree of pulmonary vascular obstruction from PE go on to develop PH with variable severity. The answer is not simply the degree of clot blockage or its persistence (Es et al., 2013; Exter et al., 2015; Klok, Dzikowska‐Diduch, et al., 2016; Morris, 2013). Development of PH requires blockage plus vasospasm and maladaptive remodeling, resulting in permanent decrease in cross‐sectional vascular area (Delcroix et al., 2016; Gopalan et al., 2017). These findings have raised the “misguided angiogenesis” hypothesis as a mechanism of CTEPH, and conversely, increased angiogenesis can prevent CTEPH by two broad mechanisms (Alias et al., 2014; Quarck et al., 2015). First, new vessels increase cross‐sectional vascular area thus decreasing pulmonary vascular resistance, and second by enhanced vascular ingrowth into thrombi hastening their resolution (Alias et al., 2014; Sharma & Lang, 2018). Angiogenesis refers to the sprouting of new vessels from existing vessels, driven by extension, and proliferation of endothelial cells. Vascular endothelial growth factor (VEGF) is the master regulator of both the growth and maintenance of pulmonary vessels, although VEGF‐driven angiogenesis is enhanced by, and may even require, other growth factors, notably PDGF (Heldin et al., 1998; Hellberg et al., 2010; Voelkel & Gomez‐Arroyo, 2014). Disruption of VEGF signaling with the VEGF receptor 2‐selective tyrosine kinase inhibitor SU5416 is well known to convert hypoxia, which alone seldom produces PH, into an animal model with a nearly 100% rate of PH (Voelkel & Gomez‐Arroyo, 2014). sGC is a heterodimeric protein which is the receptor for the important vasodilator nitric oxide (NO; 34). Binding of NO to sGC requires the presence of a heme moiety with a coordinated ferrous iron ion, Fe^2+^ (Derbyshire & Marletta, 2012). Oxidation of ferrous iron to ferric iron Fe^3+^ results in loss of the heme moiety, non‐responsiveness to NO and impaired vasodilation. Pharmacologic agonists have been developed to modulate sGC activity (Brusilovskaya et al., 2019; Priviero & Webb, 2010; Schermuly et al., 2011). These compounds behave by two distinct mechanisms. sGC *stimulators* bind to and are agonists for native heme‐containing, ferrous sGC (example, riociguat, RIO, BAY 63‐2521) while sGC *activators* bind to and are agonists for heme‐free sGC lacking its iron ion (example, cinaciguat, CINA, BAY 58‐2667; 11; Kollau et al., 2018). sGC stimulators sensitize the reduced form of sGC to endogenous and exogenous NO. On the other hand, sGC activators re‐activate oxidized or heme‐free states of sGC that have become unresponsive to NO due to disease processes involving increased oxidative stress (Evgenov et al., 2006).

We have previously described a novel rat model of chronic pulmonary embolism (CPE) that combines embolization with polystyrene microspheres and suppression of angiogenesis by post‐PE treatment with SU5416 (Neto‐Neves et al., 2017). Rats embolized with microspheres in the absence of SU5416 rarely develop chronic, progressive PH. Barium‐contrasted lung CT scans of these rats reveal extensive new pulmonary vessel growth 3–4 weeks post‐PE and a 75% increase in vascular bed volume essentially rescuing the lungs from the effects of the embolization. Significantly, when embolized rats are administered a single dose of SU5416, rats fail to show pulmonary angiogenesis by barium‐contrast CT and 95% develop severe PH, effectively modelling the human CTEPH phenotype (Neto‐Neves et al., 2017). In this current study we report on the effects of the sGC agonists RIO and CINA on pulmonary angiogenesis and reduction in chronic PH.

## 2 METHODS AND MATERIALS

### 2.1 Rats

Weighted 350 to 380‐grams male Sprague‐Dawley rats were purchased from Harlan Sprague Dawley. All rats had free access to food and water prior to use.

### 2.2 Chronic pulmonary embolism model

CPE was induced in rats as previously described by our lab with some modifications (Neto‐Neves et al., 2017). Briefly, rats were sedated with isoflurane/O_2_ under controlled inhalation conditions and PE induced by injection in the tail vein with 90 μl/100‐g body weight of 85‐micron copolymer microspheres (Thermo Fisher #184942). This dose was empirically determined to produce RVSP of ≥50 mmHg by catheterization of the right jugular vein using 2 French Millar pressure transducing catheters (Millar‐AD Instruments NA; Colorado Springs CO # SPR‐513) and the LabView2013 Pressure Precision T1600vi biometric software (National Instruments). One day after injection of microspheres rats were injected subcutaneously with 1ml/kg of SU5416 (Tocris 3037 or MedChem Express HY‐10374) dissolved in DMSO at 20 mg/ml (20 mg/kg final dose). Treatment of rats with CINA or RIO began at 7 days post‐PE for a total of 2 weeks. CINA (Tocris 6052 or BAY 582667; gifted from Bayer‐AG) or RIO (MedChem Express, #HY‐14779) was administered to rats by subcutaneous injection twice daily with 0.83 ml/kg chemical dissolved in DMSO at 3 mg/ml (2.5 mg/kg/injection final dose). Control rats received a fixed dose of DMSO at 0.3 ml/injection.

### 2.3 Terminal measurements and tissue harvests

Rats were anesthetized with continuous isoflurane inhalation. RVP measurements were made as described above. Measurements of VO_2_Max and cardiac output have been previously described (Brown et al., 2017, Watts et al., 2006, 2008). Whole blood was withdrawn into EDTA‐containing tubes and plasma separated by high‐speed centrifugation (1 min at 10,000 *g*). Lungs were extensively perfused with saline solution by cannulating the pulmonary artery with a 25‐gauge syringe needle. The left lung lobe was transferred into 10% neutral buffered formalin for fixation and the right lobes were freeze‐clamped and stored at −80°C. Hearts were excised and separated into RV and LV+septum sections, weighed for Fulton index metrics and then freeze‐clamped and stored at −80°C. In some rats, the thoracic descending aorta and pulmonary artery (main PA plus left and right branches) were carefully dissected and placed in ice‐cold saline for subsequent processing into aortic and PA rings.

### 2.4 Lung vascular volume

Lung vascular volume was quantified by high resolution compute tomography as previously described (Neto‐Neves et al., 2017). After euthanasia, heparinized saline (99‐parts 0.9% NaCl and 1‐part Heparin) was infused through the right jugular vein to flush the pulmonary circulation free of blood. A barium sulfate contrast media (BriteVu; Scarlett imaging LLC) was infused through the jugular vein catheter with the lungs inflated with air (20 mmHg) and trachea was ligated to maintain pressure. Heart and lung blocks were excised and placed on a carbon‐fiber bed and scanned with small animal high resolution micro‐CT scanner (Skyscan 1176; Bruker Biospin Corp). The micro‐CT images were acquired at 33.63 μm using three continuous bed positions to cover the entirety of the lungs, where the tube voltage, current, step‐size filter, and exposure times will be 80 kV, 313 μA, 0.9 degree/step, 0.5 mm Al filter, and 20 ms, respectively. Upon completion of the micro‐CT scans, images were reconstructed using filtered back projection via SkyScan NRecon software (version 1.6.9.4), where a Hamming filter of 0.25 (alpha) and Nyquist frequency cutoff of 100 Hz yielded an isotropic voxels with a 33.63 μm resolution. For analysis, DICOM image volumes will be imported into Analyze 12.0 (AnalyzeDirect), signal intensity normalized (i.e., [0.0, 1.0]), and tissue segmentation accomplished via segmented threshold for background+soft tissue (i.e., 0.0–0.1) and lung vasculature (i.e., 0.1–1.0). Segmented object maps are then quantified for total vascular volumes using Analyze's ROITool.

### 2.5 Aortic and pulmonary artery ring assays in Geltrex

Aortic rings were dissected almost exclusively from the aortic arch and adjacent descending thoracic aorta but in some hearts it was necessary to cut rings from the ascending aorta. Pulmonary artery rings were preferentially dissected from the left (narrower) pulmonary artery, but it was often necessary to also cut rings from the right (much wider) pulmonary artery along with left rings in the same experiment. A 96‐well cell culture plates were prepared for ring assays by applying 20 μL of cold Geltrex artificial basement membrane (Gibco‐Thermo Fisher #A1413202) per well to plates kept on ice and then centrifuging the plates for 10 min at 300 rpm and 4°C to distribute the Geltrex and eliminate air bubbles. Plates were stored at 37°C for at least 30 min to allow for gelling. Plates were then put on ice again and an additional 40 μl of Geltrex applied over the previous 20 μl. Rings were carefully cut in sterile plastic dishes containing sterile serum‐free rat endothelial cell medium (Cell Biologics, M1266) removed from dissection dishes with sterile forceps, dipped into for M1266 for cleaning, dried very carefully with sterile gauze and inserted into the ice‐cold liquid Geltrex in wells. After all rings had been placed, plates were stored at 37°C for at least 30 min for gelling. Geltrex‐embedded rings were treated with 100 μl of M1266 media containing appropriate additives, which was changed daily. At the end of 10 days rings were labeled with 3‐(4,5‐dimethylthiazol‐2‐yl)‐2,5‐diphenyltetrazolium bromide (MTT) to enhance visibility of sprouts then fixed with formaldehyde. Rings were imaged at 4x magnification using an Evos phase contrast microscope.

### 2.6 Endothelial tube assay on Geltrex

Rat pulmonary microvascular endothelial cells (PMVEC; Cell Biologics RA6011) or pulmonary artery endothelial cells (PAEC; Cell Biologics RA6059) were grown in complete M1266 medium until approximately ¼ to ½ confluent, washed with PBS and starved overnight in basal M1266 plus 0.1% serum but lacking supplemental growth factors (EGF, VEGF). Sub‐confluent cells were harvested with TrypLE trypsin (Gibco Thermo Fisher #12605093), diluted with basal M1266 basal medium (serum‐free, growth factor‐free), strained through 40‐micron cell strainers (Sigma #CLS 431750) to eliminate aggregates and collected by centrifugation. Tube assays were done in 96 well plates containing 30 μl/well pre‐aliquoted and gelled Geltrex.  Aliquots (100 μl) of cells PMVECs, 25k/well; PAECs, 20k/well) plus supplements were aliquoted on to Geltrex and tubes allowed to form overnight. Tubes were then fixed with 10% neutral buffered formalin and imaged by phase contrast microscopy. All images were enhanced equally in Image J with the following commands: Image>Adjust>Brightness/Contrast>Auto; Process>Smooth; Process>Noise>Despeckle. Angiogenic metrics of tubes were measured with the Angiogenesis Analyzer plug‐in for Image J software using default settings.

### 2.7 Study approval

All animal experiments were approved by the IACUC at Indiana University‐Purdue University Indianapolis (IUPUI), Indiana University School of Medicine, Indianapolis. The approved protocol number was 11162 assigned to Jeffrey A. Kline, MD as Principal Investigator.

## 3 RESULTS

### 3.1 CINA reduces right ventricular systolic pressure and RV damage in rats with PH caused by CPE

**FIGURE 1:**
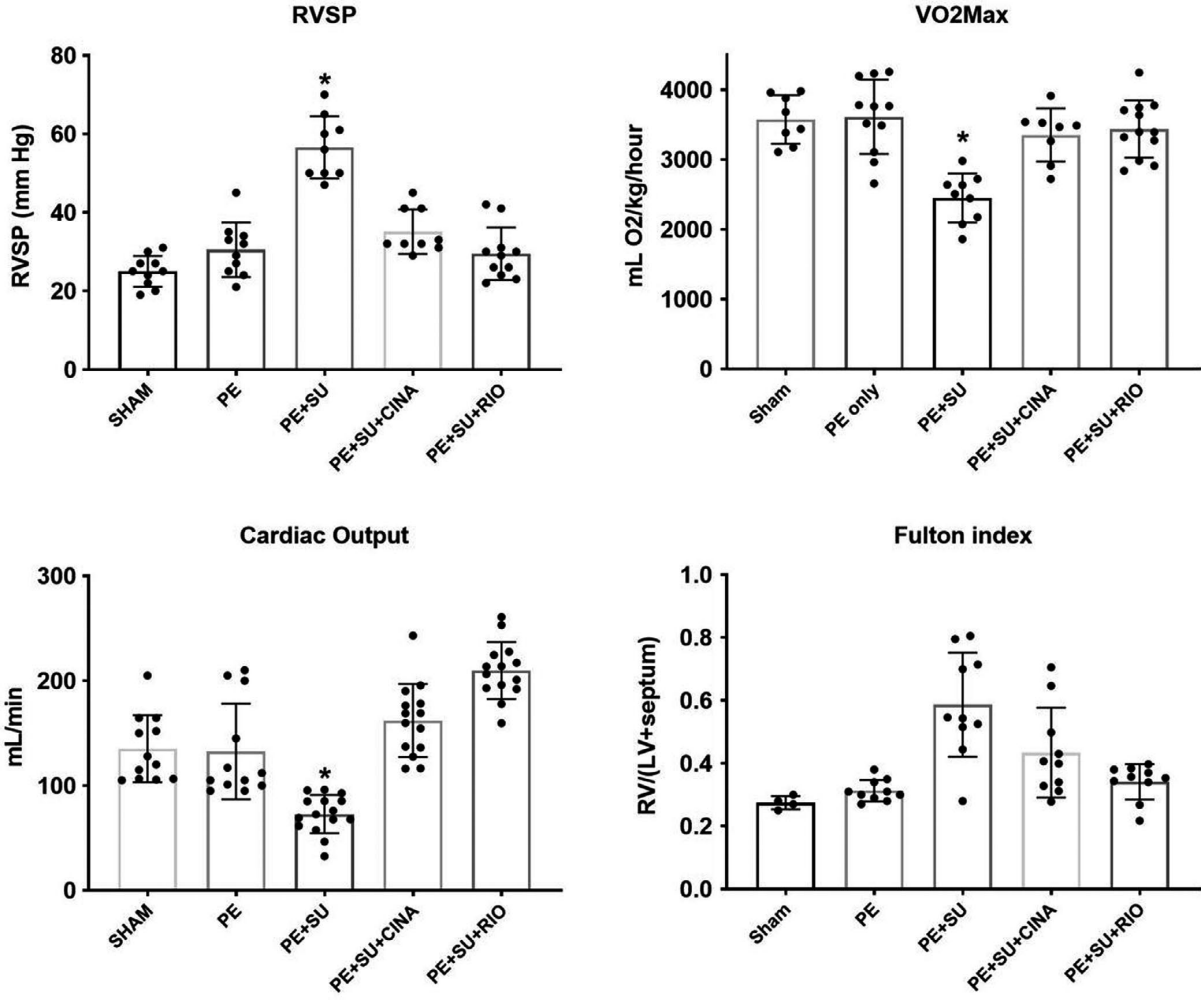
Plot of in vivo values obtained from rats. Right ventricular systolic pressure (RVSP) was measured with a micromanometer inserted via the jugular vein in anesthetized rats. VO_2_Max was the total oxygen consumption measured at peak exercise; cardiac output was measured in lightly anesthetized rats with echocardiography; the Fulton index (mass RV/[mass septum+LV]) was obtained at terminal harvest. CINA, cinaciguate; PE, pulmonary embolism; RIO, riociguate; SU, SU5416. The asterisk indicates *p *< 0.05 by Tukey's post hoc following one‐way ANOVA versus all other groups. Sample sizes are represented by dots in the figure

**FIGURE 2:**
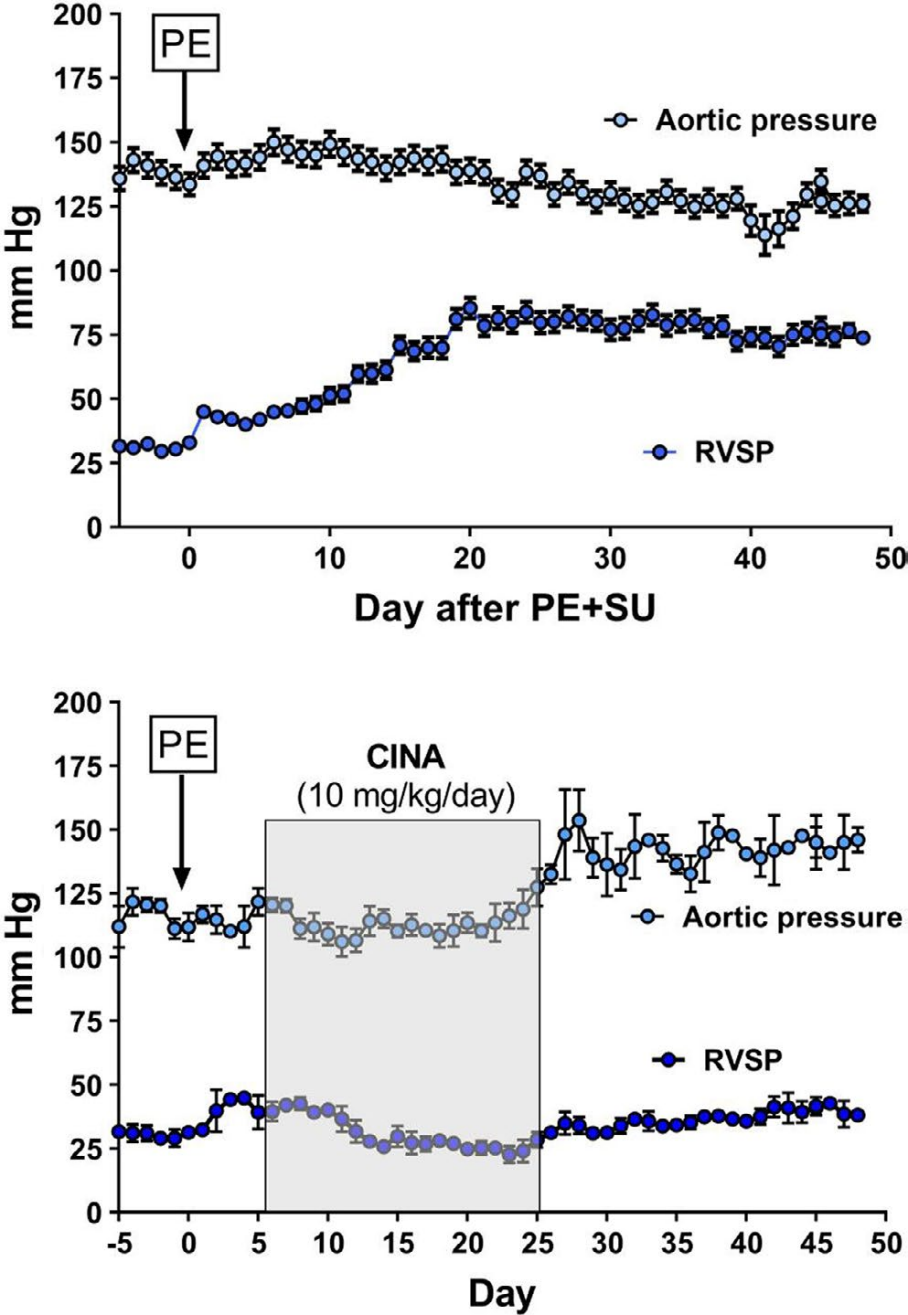
Aortic and right ventricular pressures measured over 50 days in two groups of conscious, freely moving rats, instrumented with telemetrically connected micromanometers. The top panel shows the effect of PE+SU5416 (marked by PE in box) without further treatment (*n* = 3). The bottom panel shows the effect of PE+SU5416, followed 7 days later by 10 mg/kg/day of cinaciguat, injected subcutaneously daily (*n* = 3). The data show the mean of 1 min of monitoring, taken at approximately noon each day (*x*‐axis)

Dosing of rats with SU5416 following microsphere PE resulted in elevated right ventricular systolic pressure (RVSP) at 28 days post‐PE. Treatment of CPE rats with CINA or RIO reduced RVSP to near normal values (Figure 1a), accompanied by proportional improvements in reduced VO_2_Max (1b), cardiac output (1c), and Fulton index (mass RV/mass LV+septum) (1d). Telemetric monitoring of rat RV and aortic pressures over a 7‐week time frame revealed PH development with PE+SU5416 (Figure 2 top). RVSP spiked briefly following microsphere infusion, consistent with our previously published results using an earlier PE model (Watts et al., 2006, 2008; Zagorski et al., 2003, 2007). RVSP increased linearly for approximately 3 weeks and then remained elevated for 7 weeks post‐PE. Left ventricular pressure was not significantly altered. Delayed administration of CINA to CPE rats reversed the acute rise in RVSP following microsphere infusion (Figure 2 bottom, days 0–13) and normalized RVSP during the entire 7‐week monitoring period even after discontinuation of CINA dosing at day 20. Curiously, LVSP increased near the end of the CINA treatment period and continued to increase until reaching a variable plateau of ~130–150 mm pressure. Telemetric monitoring was not done with RIO‐treated rats.

### 3.2 CINA or RIO treatment rescues pulmonary angiogenesis

**FIGURE 3:**
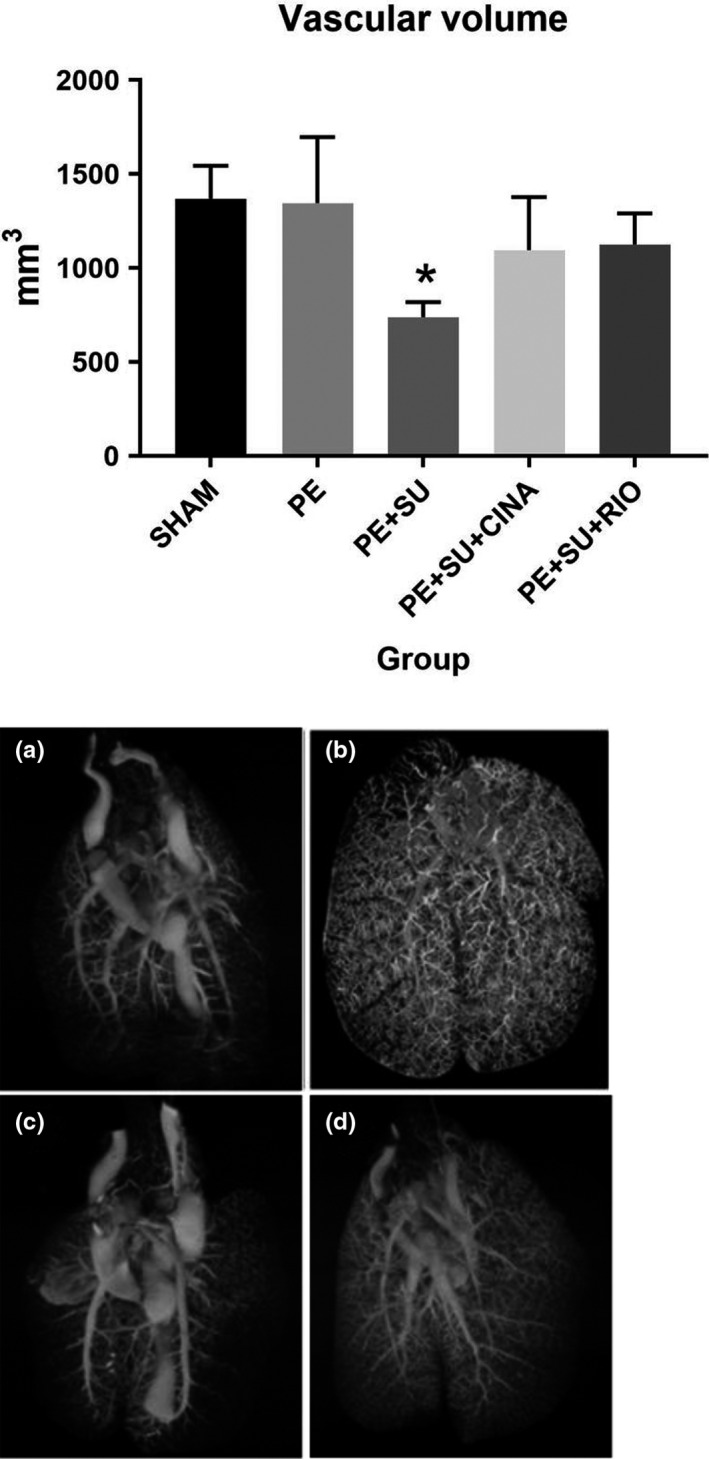
Post‐mortem computerized tomographic measurement of pulmonary vascular volumes with representative images (a = Sham, b = PE, c = PE+SU5416, d = PE+SU5416+CINA; no image available for PE+SU+RIO). CINA, cinaciguat; PE, pulmonary embolism with microspheres alone; RIO, riociguat. The asterisk indicates significance at *p *< 0.05 from Tukey's post hoc following one‐way ANOVA, versus all other groups

As previously reported, rats with CPE have increased pulmonary vascular volume as determined by post‐mortem barium‐contrast CT imagery followed by 3‐D reconstruction for volume determination, while rats also receiving SU5416 had greatly reduced angiogenesis (Neto‐Neves et al., 2017). The addition of a 2‐week course of CINA or RIO treatment to CPE rats resulted in significantly increased pulmonary vascular volume (Figure 3 top). This effect was evident in CT imaging of barium‐contrasted lungs in rats treated with CINA (Figure 3d vs. 3c). Despite the ability to quantify volume, because of technical problems with the 2‐D to 3‐D image assembly process, we were not able to obtain high quality images of lungs from RIO‐treated rats. Taken with the salutary effect of CINA on RV function, these data implied a protective effect of increased angiogenesis following PE which was inhibited by SU5416 and rescued by activation/stimulation of sGC by CINA and RIO.

**FIGURE 4:**
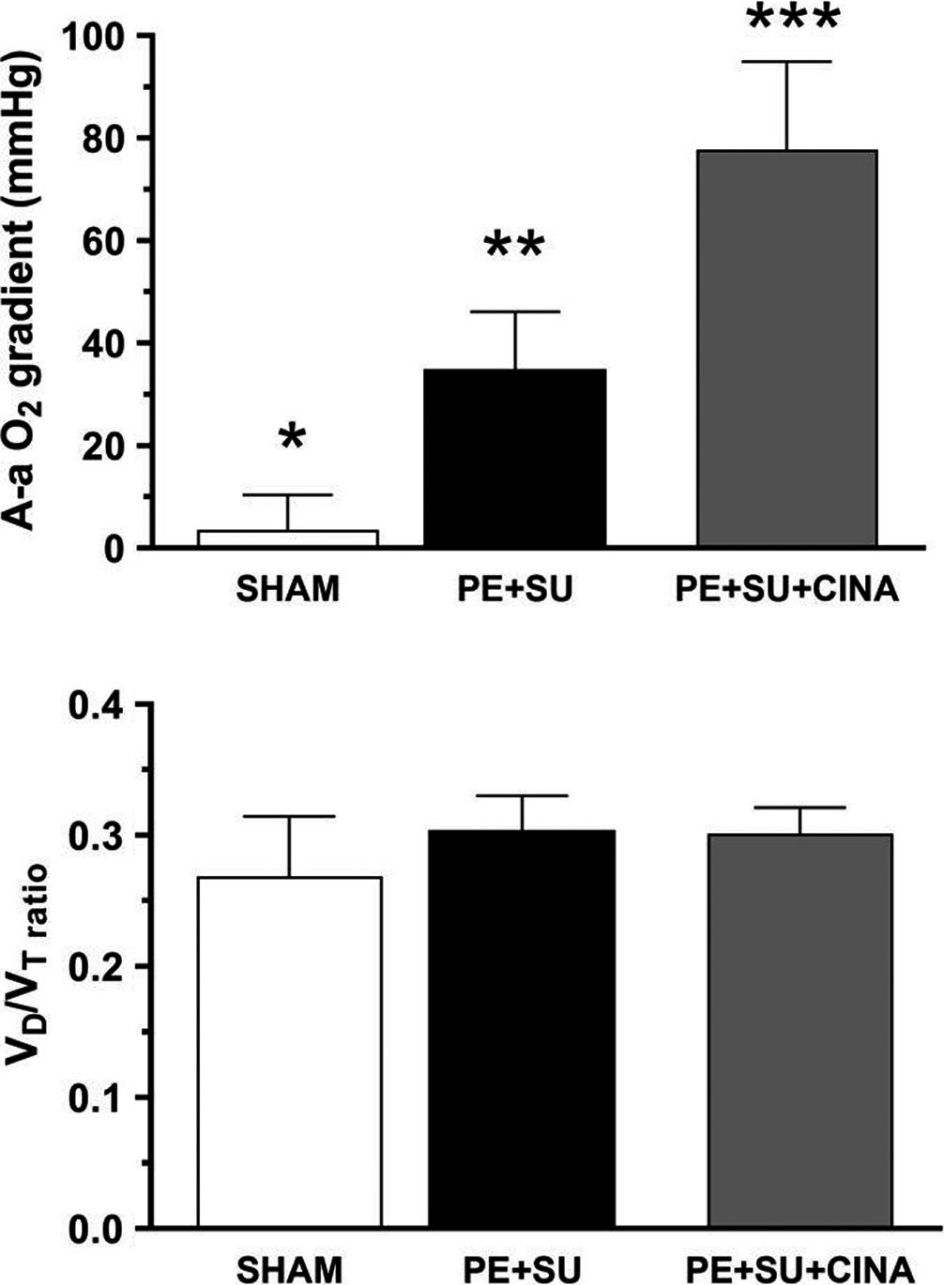
Gas exchange parameters from rats (*n* = 5 per group) treated with Sham conditions (SHAM), pulmonary embolism (PE)+SU5416 (SU)+cinaciguat (CINA), or PE+SU5416 alone. The top panel shows the alveolar‐arterial difference in partial pressure of oxygen A‐aO_2_ gradient = (0.21[Patm‐47]−PetCO_2_/0.8)−PaO_2_. The bottom panel shows the alveolar deadspace ratio (*V*_D_/*V*_T_ = PetCO_2_/(PaCO_2_−PetCO_2_). Significance determined by Tukey's post hoc following ANOVA. *p < 0.05 versus PE+SU and PE+SU+CINA, **p < 0.05 versus SHAM and PE+SU+CINA, ***p < 0.05 versus SHAM and PE+SU

**FIGURE 5:**
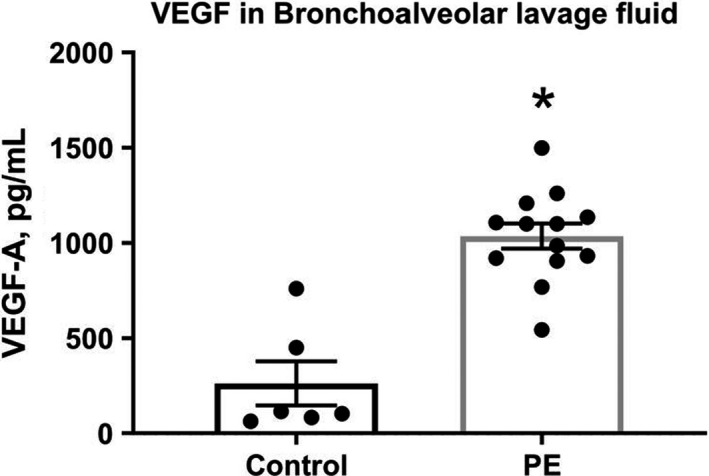
Concentrations of vascular endothelial growth factor (VEGF) alpha in bronchoalveolar lavage fluid taken 24 h after induction of microsphere pulmonary embolism (PE) (as described in methods) versus control. * indicates *p *< 0.05 by unpaired *t*‐test

To better understand the function of these new vessels in gas exchange, in a subset of anesthetized, mechanically ventilated animals, we obtained arterial blood via femoral artery cut down and cannulation, and measured partial pressures of oxygen and carbon dioxide, while simultaneously measuring the exhaled end‐tidal CO_2_. These measurements allowed calculation of the arterial‐alveolar oxygen gradient (arterial PO_2_‐alveolar CO_2_) and alveolar dead space estimate from the Severinghaus equation ([PaCO_2_‐PetCO_2_]/PaCO_2_). These data in Figure 4 suggested an increase in shunt fraction without an increase in alveolar dead space, again supporting the hypothesis of intrapulmonary vascularization out of proportion to alveolar volume. Furthermore, the bronchoalveolar lavage fluid (BALF) concentration of VEGfA (measured by enzyme‐linked immunoassay) was significantly elevated in PE rats versus controls using ELISA measurements (Figure 5).

### 3.3 Aortic and PA explants retain their angiogenic phenotypes when cultured as rings in Geltrex basement membrane preparation

**FIGURE 6:**
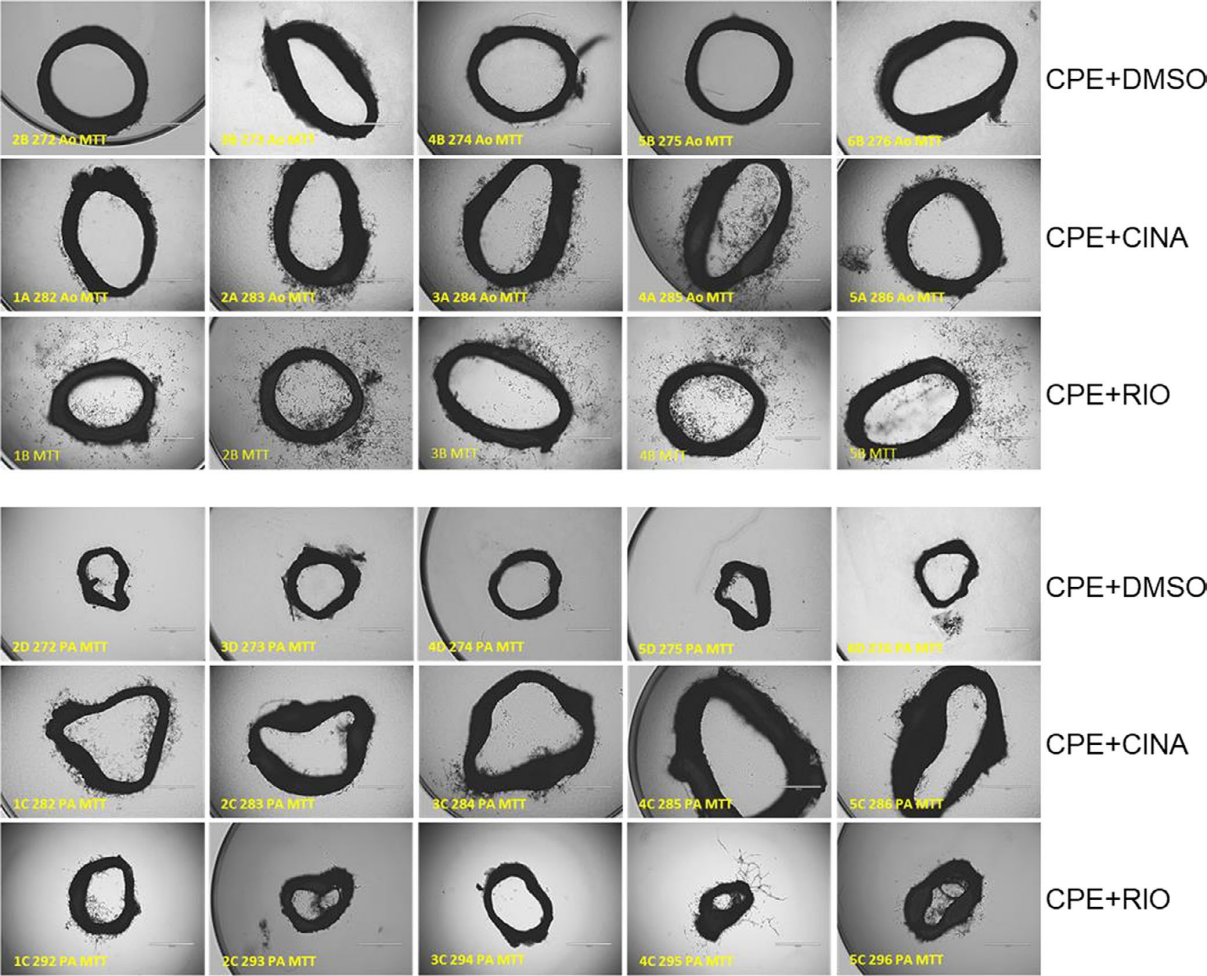
Phase‐contrast images of aortic (Ao; top 3 lines) and pulmonary artery (PA; bottom 3 lines) rings. Aortas and pulmonary arteries were dissected from rats 4 weeks after treatments to produce CPE. Rings were embedded in Geltrex and over‐laid with basal M1266 medium plus glutamine, antibiotic‐antimycotic, and 0.1% FBS but lacking growth factors (EGF, VEGF). After 10 days rings were labeled with MTT to enhance visibility of sprouts and fixed with 2% formaldehyde. Representative images are shown for five rats each from groups of 8–10 CPE rats treated with either cinaciguat (CINA), RIO, or DMSO. Aortas and PAs were obtained from the same rat in each treatment group

Aortas and pulmonary arteries were dissected from CPE rats treated with either DMSO (control), CINA, or RIO and assayed for angiogenic phenotype using Geltrex ring assays. These data are shown in Figure 6 for aortic (top panels) and PA rings (bottom panels). Images are shown for rings which were metabolically labeled with MTT to enhance the visibility of otherwise faint angiogenic sprouts. Rings obtained from CPE rats treated with either CINA or RIO had more sprout formation than rings from control rats receiving only DMSO. Aortic rings from rats treated with CINA showed the largest increase in sprout formation relative to controls. These ex vivo data were consistent with the enhanced vascularization of pulmonary vessels seen in vivo by barium‐enhanced CT. The data also indicate that the angiogenic phenotype of vessels exposed to CINA and RIO in rats was maintained for at least 1‐week post treatment in vivo and that the phenotype was maintained through at least 10 days of ex vivo culture.

### 3.4 SU5416 inhibits endothelial tube formation on Geltrex; rescue by CINA

**FIGURE 7:**
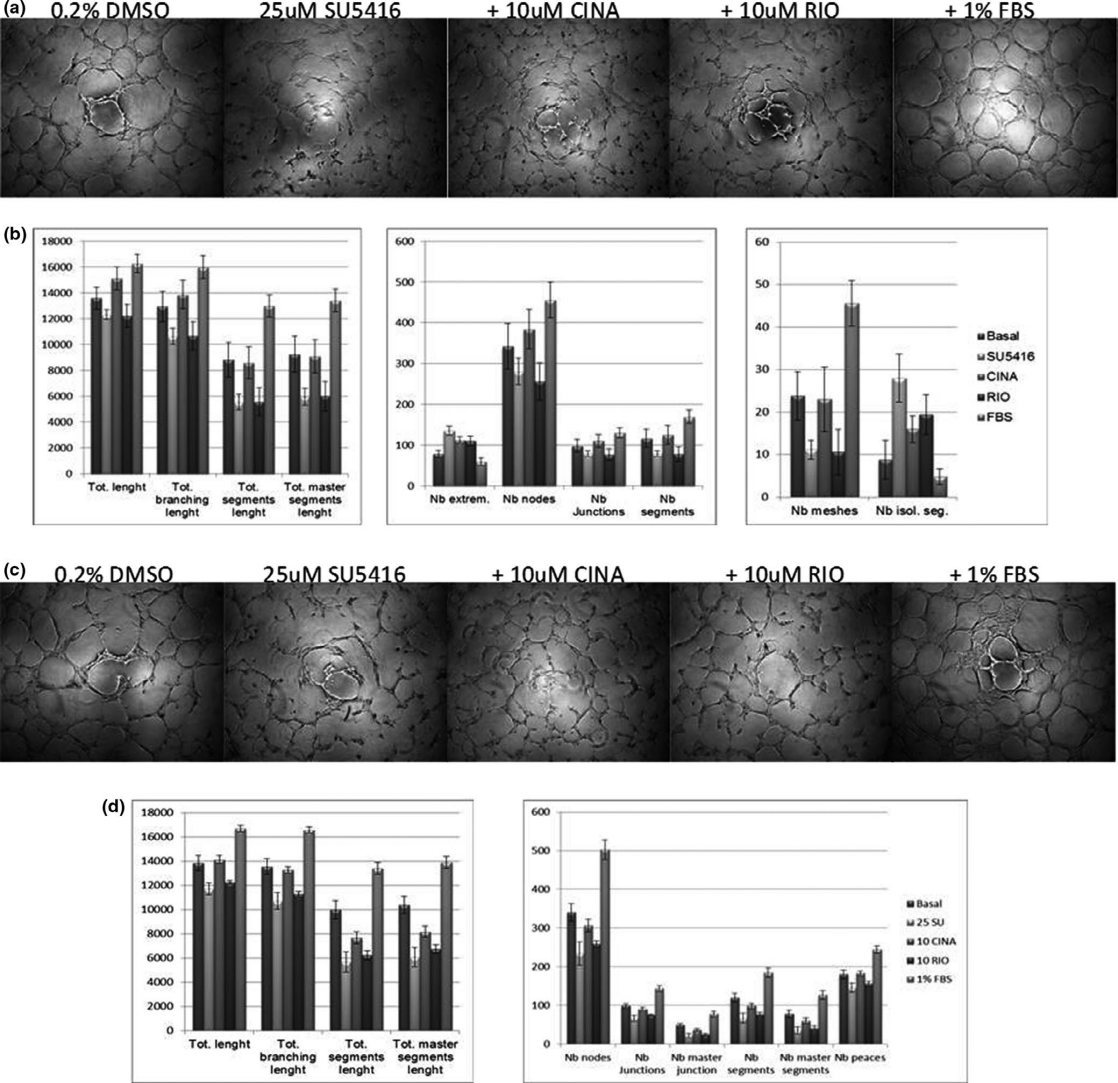
Geltrex tube assays of rat endothelial cells treated with SU5416 plus cinaciguat (CINA) or riociguat (RIO). Panel a: Phase‐contrast images of representative wells from triplicate assays of pulmonary microvascular endothelial cells (PMVECs) (25k/well) treated with the indicated supplements. Control cells were incubated with M1266 basal medium containing glutamine and antibiotic‐antimycotic mix without growth factors or serum. DMSO was added at a concentration of 0.2% in control samples. SU5416, CINA, and RIO dissolved in DMSO were added to M1266 basal medium at the indicated concentrations. Panel b: Angiogenesis Analyzer metrics of PMVECs treated in Panel a. Representative metrics in which SU5416+CINA treatments were significantly different from SU5416 alone are shown (*p *< 0.05, ANOVA with Tukey's pairwise test). Panels c and d, same as a and b except PAECs (20k/well)

A preliminary dose‐response experiment indicated that 25 μM SU5416 was required to inhibit PMVEC or PAEC tube formation of on Geltrex (data not shown). Some disruption of tube structures was observed at SU5416 concentrations of 0.25 and 2.5 μM, but these were not statistically significant when images were tested by the Angiogenesis Analyzer (AA) plug‐in for Image J (data not shown). Phase‐contrast images of endothelial tube networks formed on Geltrex are shown in Figure 7a,c. The disruptive effect of SU5416 on tube networks by both PMVECs and PAECs is evident in the DMSO control versus SU5416 images qualitatively and quantitatively in Figure 7a–d. Co‐treatment with 10 μM CINA, but not 10 μM RIO, improved tube networks in both cells. Image J quantitates 20 metrics of tube formation with varying amounts of sensitivity (ability to distinguish small differences in tube network complexity). SU5416 disrupted 10 and 19 of these metrics for PMVECs and PAECs, respectively (not shown). When cells were co‐treated with SU5416 plus CINA, 10 metrics each were improved for PMVECs and PAECs, while RIO improved only one metric for PMVECs and none for PAECs (not shown). Quantitation of the SU5416, CINA and RIO treatments by Image J AA is shown in Figure 7b,d for select AA metrics for which SU5416+CINA was significantly improved versus SU5416 alone. Increasing the RIO concentration in this assay from 10 uM to 100 uM did not result in improved tube formation (data not shown).

## 4 DISCUSSION

Treatment with the sGC stimulator and activators CINA and RIO both ameliorated the development of the CTEPH phenotype in rats with CPE accompanied by in vivo and in vitro evidence of enhanced angiogenesis. Metrics of right ventricular function were improved by both compounds, including RVSP, exercise tolerance, and right ventricular hypertrophy. These results support the use of sGC modulators to ameliorate the progression from CTED to CTEPH in human patients with PE. The beneficial effects of sGC agonists were accompanied by increased pulmonary angiogenesis in vivo, implying a causal link. This linkage was supported by several lines of evidence. First, aortic and pulmonary artery rings derived from rats treated with CINA or RIO had higher angiogenic potential when cultured in Geltrex than rings from rats with CPE but no CINA or RIO treatment. Second, CINA, but not RIO, improved endothelial tube formation on Geltrex basement membrane formulation in pulmonary endothelial cells treated with SU5416. Both of these results suggest that sGC agonists “rescue” the angiogenic potential of pulmonary endothelial cells from the VEGF/VEGF‐R2‐signaling inhibition caused by SU5416. It is unclear why RIO did not rescue endothelial tube formation on Matrigel, even at an elevated dose of 100 μM, which was cytotoxic (data not shown). RIO was active in stimulating sGC activity in whole tissues extracts of rat lung with cGMP production as an endpoint (data not shown). This contrasts with the pro‐angiogenic effect RIO had on rats in vivo and the increased angiogenesis we observed in ex vivo aortic and PA rings taken from RIO‐treated rats (Figure 6).

We propose that angiogenesis occurs from the normal healing response to persistent PE, as is the case with the present model, which occurs in two phases: acute inflammation followed by angiogenesis (Ridiandries et al., 2016). Acute PE in humans and animals produces a robust inflammatory response within hours, characterized by increase in transcription and expression of chemokines in the lungs and increased circulating concentrations of interleukins, followed by cellular infiltration in the lung and heart (Nakos et al., 1998; Stewart et al., 2015; Zagorski et al., 2003, 2007). Our data in rats show that the lung switches from an inflammatory phenotype to an angiogenic phenotype starting a few days after PE, and that disruption of VEGF signaling with the VEGF‐R2 inhibitor SU5416, reliably causes severe PH post‐PE (Neto‐Neves et al., 2017). Angiogenesis refers to the sprouting of new vessels from existing vessels, driven by extension and proliferation of ECs (Stapor et al., 2014). The growth factor VEGF has been called a master regulator of both the growth and maintenance of pulmonary vessels, although VEGF‐driven angiogenesis may even require other cytokines and growth factors, notably PDGF (Heldin et al., 1998; Hellberg et al., 2010; Voelkel & Gomez‐Arroyo, 2014). In animal models, VEGF is increased in experimental thrombi and contributes to clot resolution (Waltham et al., 2000, 2003). SU5416 is well known to cause PH together with ambient hypoxia (Voelkel & Gomez‐Arroyo, 2014). The role of disrupted VEGF signaling as a cause of PH post‐PE remains largely hypothetical. About 20% of humans have alternative splicing that alters VEGF signaling, but the epidemiological understanding of genetic deficiencies in VEGF signaling axis related to CTEPH is nascent. As an example, alternative splicing of the VEGF decoy receptor VEGF‐R1, sometimes called fms‐related tyrosine kinase (flt‐1), results in a soluble variant (s‐Flt1) that can sequester VEGF and decrease its activity (Kendall & Thomas, 1993). In small studies, patients with CTEPH had elevated circulating concentrations of s‐Flt1 (Saleby et al., 2017; Tiede et al., 2015). The role of antiangiogenic splice variants in the VEGF‐A protein (the VEGF_xxx_b variants) remain poorly characterized in CTEPH (Suzuki et al., 2016). CTEPH patients have elevated blood concentrations of VEGF, and increased expression of angiostatic receptor CXCR3, especially in distal lung arteries (Saleby et al., 2017; Zabini et al., 2012). Lung tissues from CTEPH patients demonstrate increased VEGF in regions of lung and in thrombus that are dense in new vascular growth, but not in fibrotic areas (Bochenek et al., 2017). Taken together, available data suggest that many patients with CTEPH have either inherited or acquired defects in VEGF signaling, although it is clear neither VEGF’s action nor its disruption is the sole mediator of either the normal angiogenic response or progression to PH, respectively.

Guanylate cyclase modulators may overcome inhibition of angiogenesis produced by the combination of PE and VEGF antagonism at the post‐receptor level. At the subcellular level, VEGF‐R2 is the primary receptor tyrosine kinase (RTK) responsible for angiogenesis, and it activates the cytosolic kinases ERK‐1 and ‐2, which have multiple effects. When phosphorylated via VEGF‐R2 signaling, the ERK1/2 isoforms increase endothelial cell growth, proliferation, survival, and phosphorylation of PFKFB3 (Novellasdemunt et al., 2012, 2013), the key regulator of glycolytic flux. This is important because glycolysis supplies cytosolic ATP necessary for endothelial pseudopod formation and tip cell extensions required for new vessel growth (Stapor et al., 2014). ERK1/2 phosphorylation can also be induced by increases in cellular cGMP mediated by the enzyme soluble guanylate cyclase (sGC) (Pyriochou et al., 2006). These data formed the rationale for the hypothesis that sGC modulation could increase angiogenesis in a tandem model of CTEPH.

## 5 CONCLUSIONS

In rats with chronic pulmonary hypertension produced by PE followed by treatment with the RTK inhibitor SU5416, sGC stimulation and activation prevents subsequent PH, coincident with increased pulmonary angiogenesis. These data suggest a role for pharmacological sGC modulation in the acute post‐PE period to reduce probability of the chronic PH phenotype.

## CONFLICT OF INTEREST

CINA was provided as an unrestricted in‐kind donation from Bayer. The authors have no other financial conflict to report.

## AUTHOR CONTRIBUTIONS

John Zagorski co‐conceived the work, collected and analyzed the data, and wrote the first draft of the manuscript. Evandro Neto‐Neves developed the model system, collected data, and edited the manuscript, Nathan J. Alves provided technical and scientific direction and edited the manuscript. Amanda J. Fisher performed surgeries, collected data, and edited the manuscript and Jeffrey A. Kline co‐conceived the work, obtained funding, collected and analyzed the data, and co‐wrote the manuscript.
